# Supplementary material for: Quantitative CT imaging features for COVID-19 evaluation: The ability to differentiate COVID-19 from non- COVID-19 (highly suspected) pneumonia patients during the epidemic period
Source: PLoS One. 2022 Jan 13;17(1):e0256194. doi: 10.1371/journal.pone.0256194 (PMC8758079; doi:10.1371/journal.pone.0256194)
Supplement: S1 File — (PDF) [file pone.0256194.s001.pdf]

Supporting information

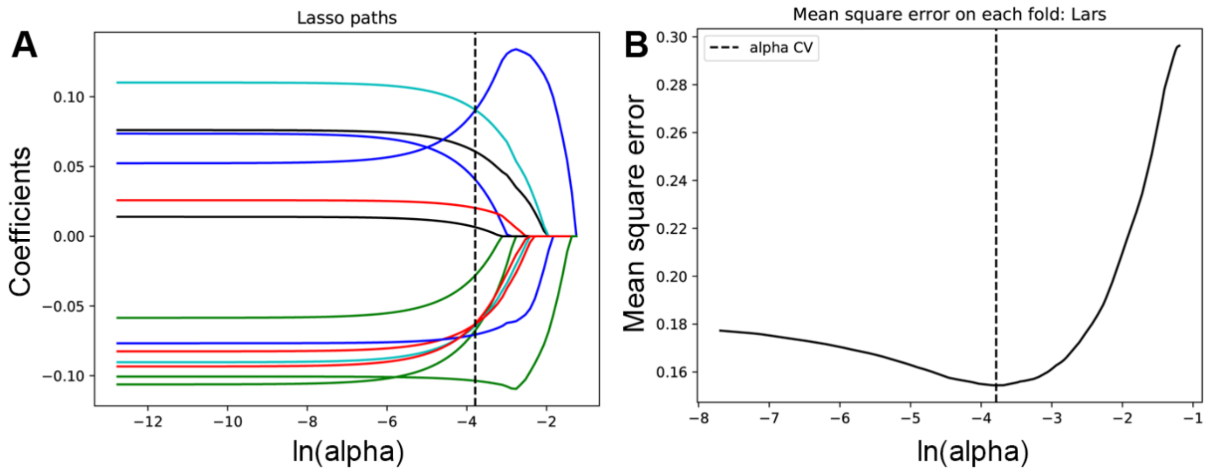

S1 Fig. The procedure of radiomic feature selection using the least absolute shrinkage and selection operator (LASSO) binary logistic regression model. （A）Parameter (alpha) is tuned in the LASSO model using 10-fold cross-validation via minimum mean square error criteria. A coefficient profile plotted against the log (alpha). Vertical line was drawn at the value elected using 10-fold cross-validation, where optimal alpha resulted in nonzero coefficients. (B) Mean square error on each fold was drawn versus log (alpha).

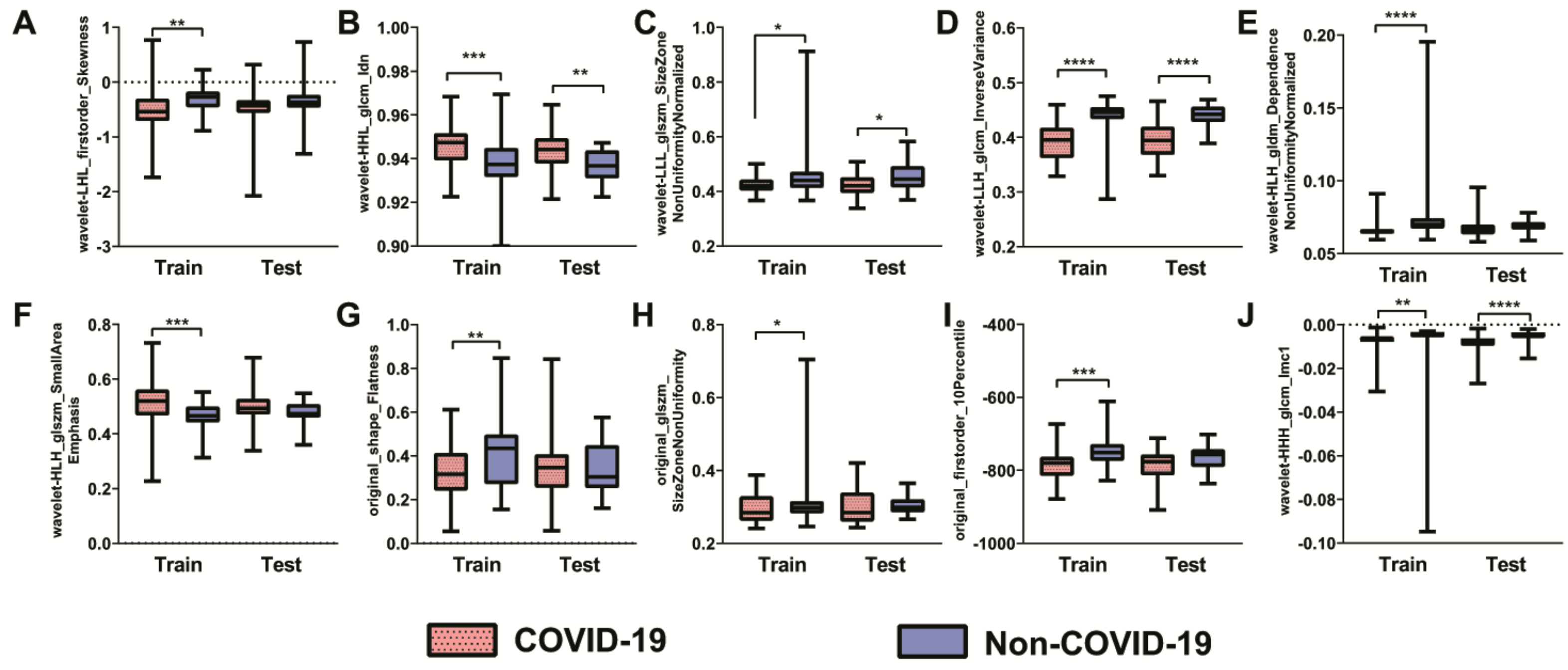

S2 Fig. The selected radiomics features distribution of Non-COVID-19(purple box) and COVID-19(pink box) groups in the training and testing sets.

Statistically significant level: *P<0.05， **P<0.01， ***P<0.001, **** P<0.0001

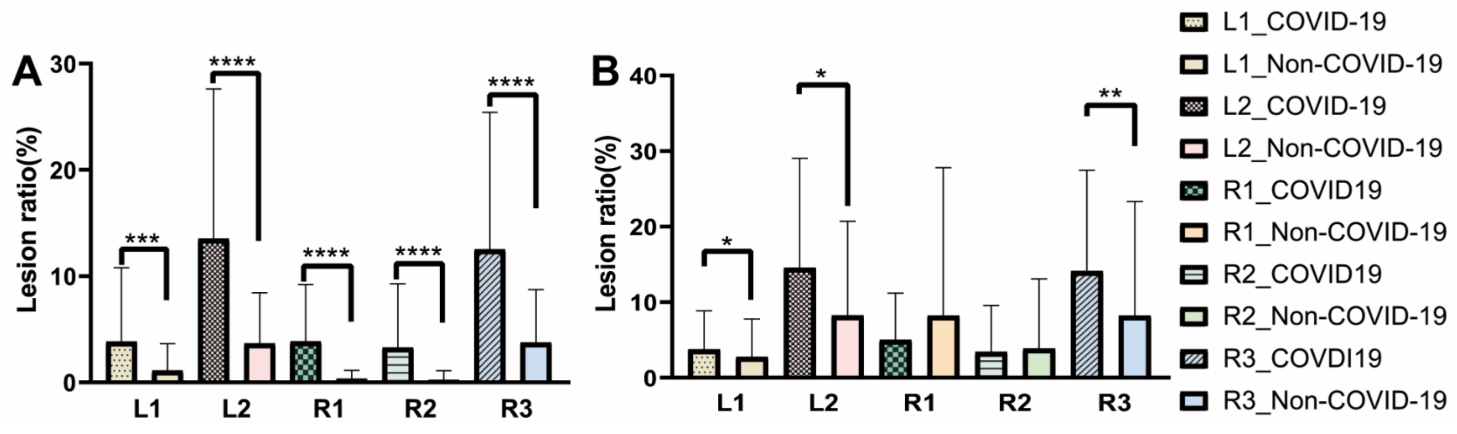

S3 Fig. The distribution of lesion volume ratio of COVID-19 and Non-COVID-19 in five lung lobes. L1: upper left lung lobe; L2: bottom left lung lobe; R1: upper right lung lobe; R2: middle right lung lobe; R3: bottom right lung lobe. (A) Training set; (B) Testing set.

Statistically significant level: *P<0.05， **P<0.01， ***P<0.001, **** P<0.0001

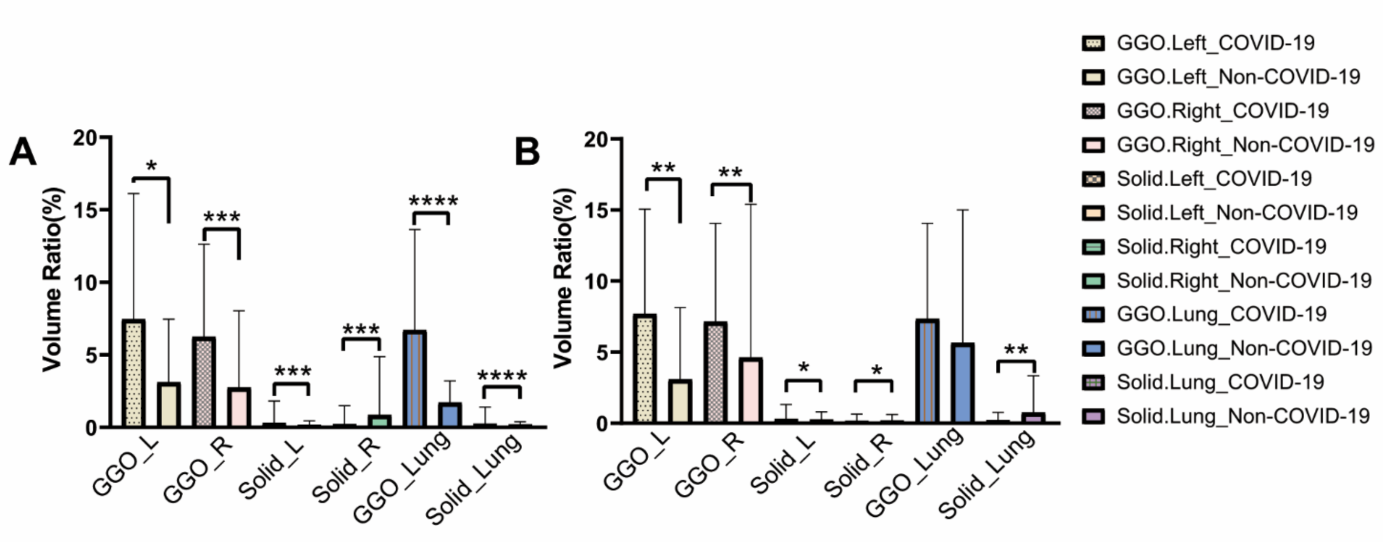

S4 Fig. The distribution of part-solid and solid volume ratio in COVID-19 and Non-COVID-19 and in left, right or the whole lung. L: Left; R: Right. (A) Training set; (B) Testing set.

Statistically significance level: *P<0.05, **P<0.01, ***P<0.001, **** P<0.0001

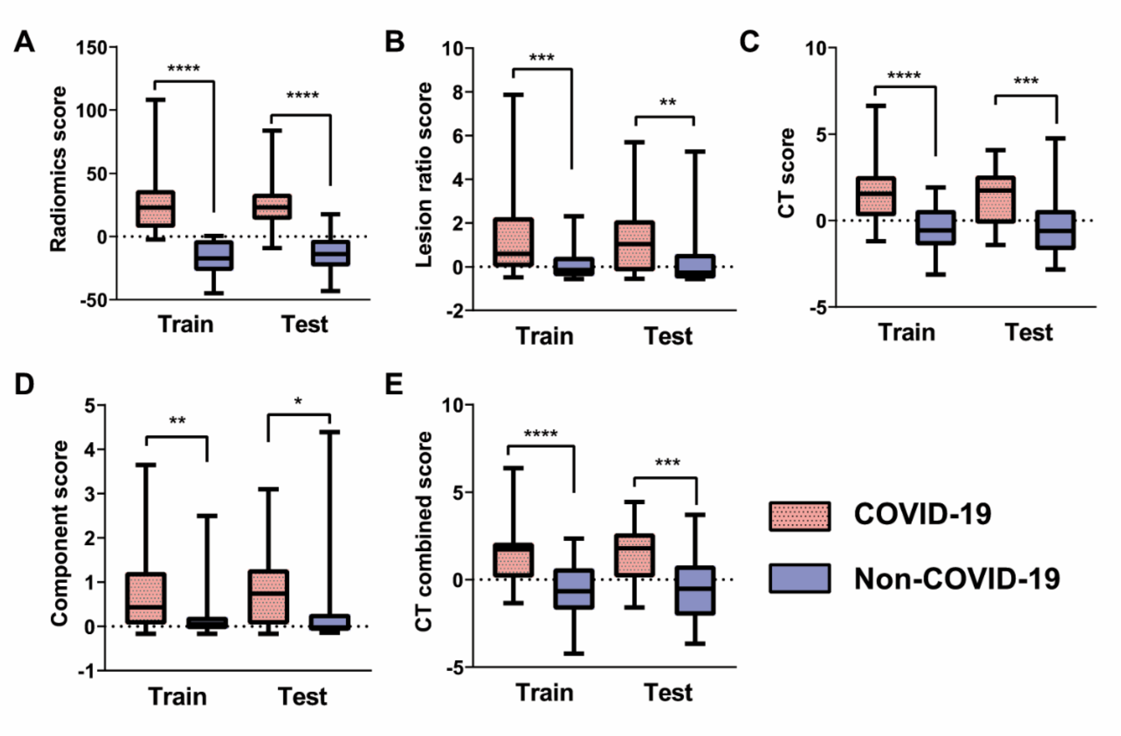

S5 Fig. The classification score of each model for Non-COVID-19 (purple box) and COVID-19 (pink box) groups in training and testing sets. (A) Model A: radiomics model; (B) Model B: lesion volume ratio model; (C) Model C: lesion score model; (D) Model D: lesion component ratio model; (E) Model E: combined model using lesion score and component ratio simultaneously.

Statistically significance level: *P<0.05, **P<0.01, ***P<0.001, **** P<0.0001

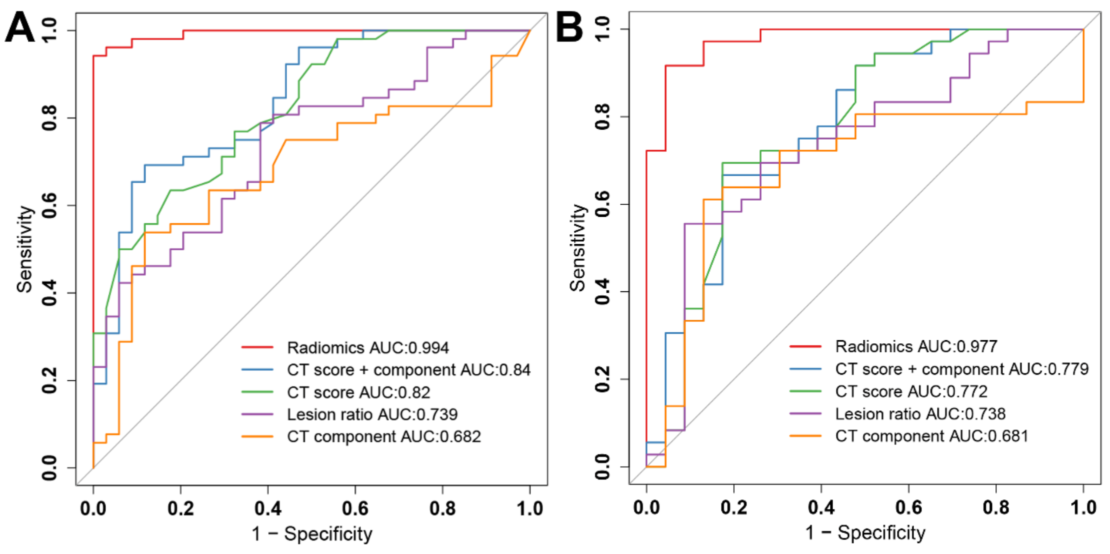

S6 Fig. The ROC curves of five models in the training (A) and testing sets (B).

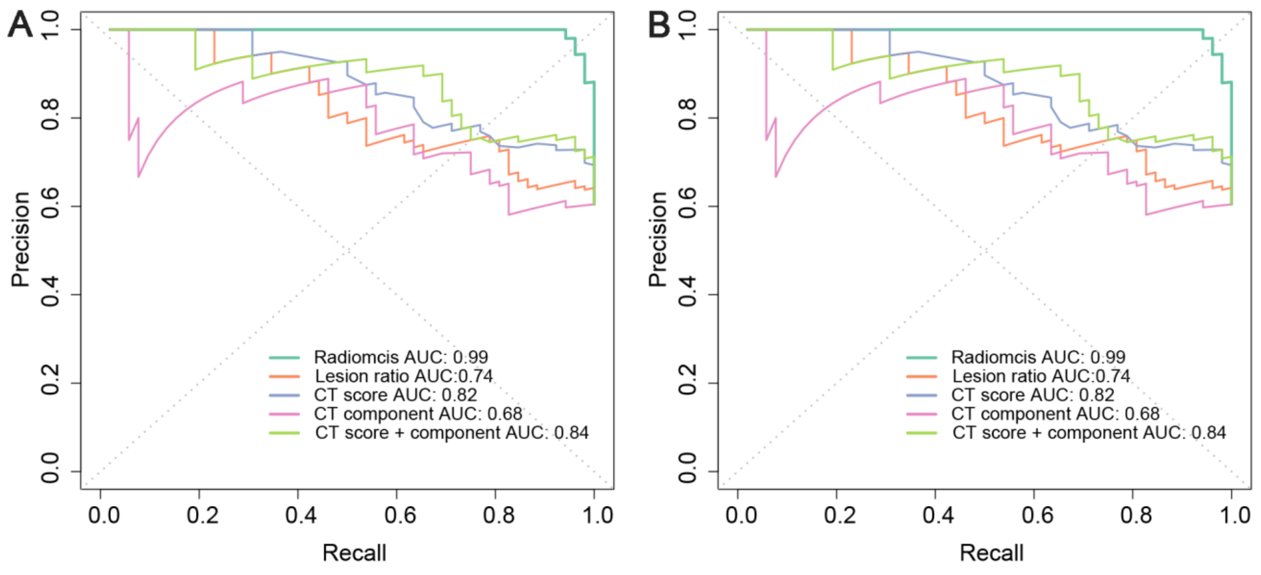

S7 Fig. The Precision-Recall (PR) curve of each model in the training (A) and testing (B) sets. The area under the PR curve for each model was labeled in the legend.

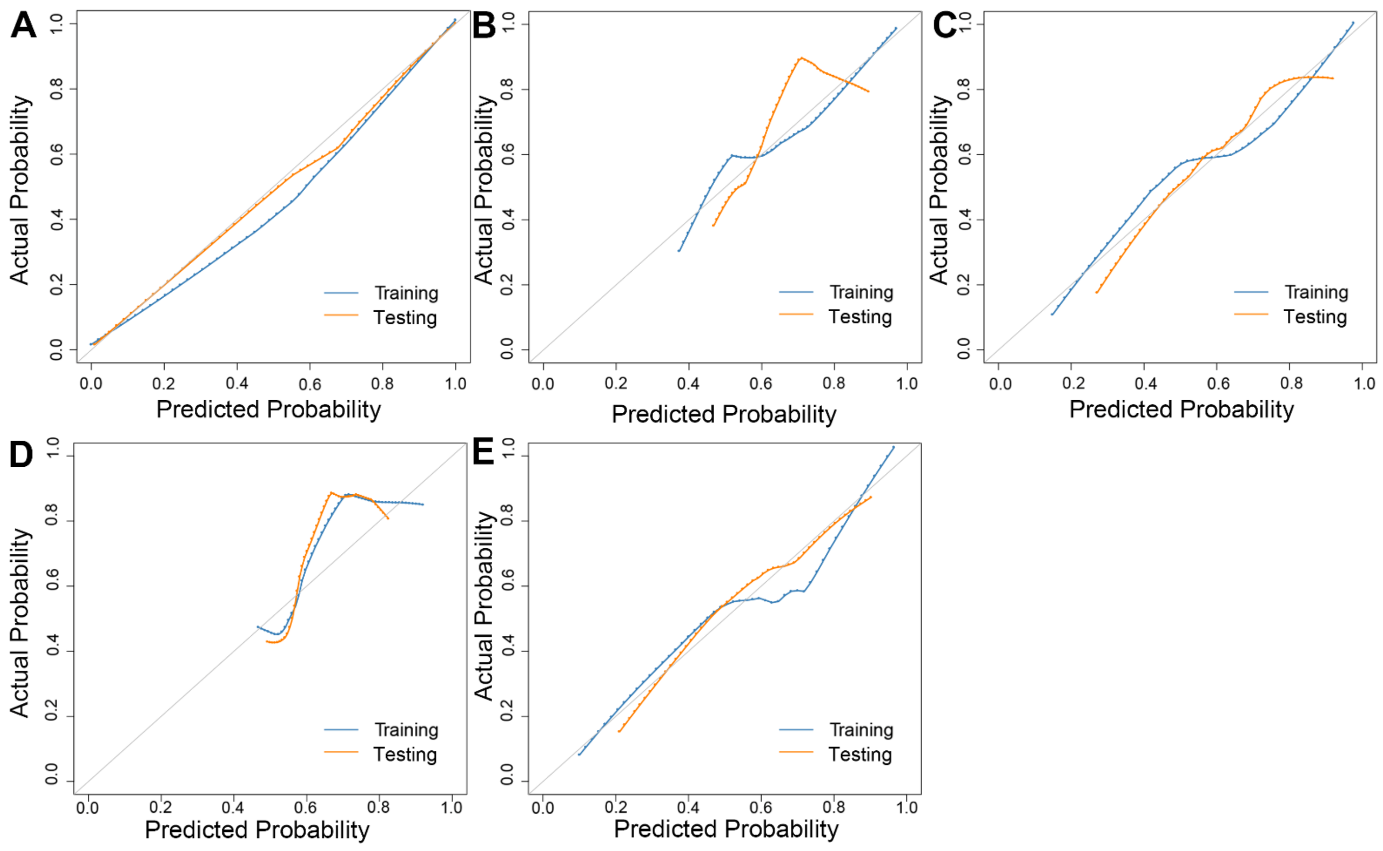

S8 Fig. The calibration curves of different models. (A) Model A: radiomics model; (B) Model B: lesion volume ratio model; (C) Model C: lesion score model; (D) Model D: lesion component ratio model; (E) Model E: combined model using lesion score and component ratio simultaneously.

Table S1 The selected radiomics features of Non-COVID-19(NC) and COVID-19 groups in the training and testing sets.

	Training set			Testing set		
Radiomics features	NC	COVID-19	P-value[a]	NC	COVID-19	P-value
wavelet-LHL_firstorder_Skewness	-0.27(-0.45, -0.18)[b]	-0.54(-0.70, -0.31)	0.002	-0.37(-0.46, -0.23)	-0.43(-0.55, -0.34)	0.128
wavelet-HHL_glcm_Idn	0.94(0.93, 0.94)	0.95(0.94, 0.95)	<0.001	0.94±0.01[c]	0.94±0.01	0.003
wavelet-LLL_glszm_SizeZoneNonUniformityNormalized	0.44(0.41, 0.47)	0.42(0.40, 0.44)	0.019	0.46±0.06	0.43±0.04	0.028

wavelet-LLH_glcm_InverseVariance	0.45(0.43, 0.45)	0.40(0.36, 0.42)	<0.001	0.44(0.43, 0.46)	0.39(0.37, 0.42)	<0.001
wavelet-HLH_gldm_DependenceNonUniformityNormalized	0.07(0.07, 0.07)	0.07(0.06, 0.07)	<0.001	0.07(0.07, 0.07)	0.07(0.06, 0.07)	0.058
wavelet-HLH_glszm_SmallAreaEmphasis	0.47(0.44, 0.50)	0.52(0.47, 0.56)	0.001	0.47(0.46, 0.50)	0.49(0.47, 0.53)	0.054
original_shape_Flatness	0.43(0.27, 0.49)	0.32(0.24, 0.41)	0.004	0.30(0.26, 0.45)	0.35(0.26, 0.41)	0.913
original_glszm_SizeZoneNonUniformity	0.30(0.28, 0.31)	0.28(0.26, 0.33)	0.147	0.30(0.29, 0.32)	0.28(0.26, 0.33)	0.171
original_firstorder_10Percentile	-751.50(-773.04, -730.50)	-780.00(-812.95, -763.90)	<0.001	-757.00(-789.60, -743.80)	-776.00(-811.40, -757.00)	0.069
wavelet-HHH_glcm_Imc1	-0.00(-0.01, -0.00)	-0.01(-0.01, -0.01)	0.005	-0.00(-0.01, -0.00)	-0.01(-0.01, -0.01)	<0.001

a. Statistically significant level: $P < 0.05$

b. The variables with abnormal distribution were depicted by median (interquartile range, IQR)

c. The variables with abnormal distribution were depicted by mean ± SD.

Table S2 The selected lesion ratio features of Non-COVID-19 (NC) and COVID-19 groups in the training and testing sets.

	Training set			Testing set		
Lesion ratio features	NC	COVID-19	P-value[a]	NC	COVID-19	P-value[a]
Lesion volume ratio (right-bottom lobe)	2.00(0.00, 6.31)	8.85(3.39, 18.08)	<0.001	1.10(0.00, 5.14)	10.30(2.61, 23.44)	0.001
Lesion volume ratio (left-bottom lobe)	2.80(0.00, 9.09)	8.80(2.88, 19.29)	0.001	0.70(0.00, 8.90)	11.70(1.64, 23.51)	0.001
GGO volume ratio (left lung)	1.95(0.00, 3.90)	4.60(1.00, 10.00)	0.014	1.10(0.02, 3.80)	7.00(1.45, 10.78)	0.01
GGO volume ratio (right lung)	1.20(0.00, 2.71)	4.00(1.00, 10.27)	0.001	1.40(0.08, 3.80)	5.00(1.72, 11.10)	0.01
Upper-left lung score			0.013[b]			0.02
0	18(52.94 %)	11(21.15 %)		12(52.17 %)	7(19.44 %)	
1	11(32.35 %)	30(57.69 %)		9(39.13 %)	19(52.78 %)	
2	5(14.71 %)	9(17.31 %)		2(8.70 %)	10(27.78 %)	
3	0(0.00 %)	2(3.85 %)		-	-	
Bottom-left lung score			<0.001[b]			0.015[b]
0	11(32.35 %)	2(3.85 %)		7(30.43 %)	1(2.78 %)	
1	11(32.35 %)	15(28.85 %)		8(34.78 %)	11(30.56 %)	
2	12(35.29 %)	24(46.15 %)		7(30.43 %)	16(44.44 %)	
3	0(0.00 %)	9(17.31 %)		1(4.35 %)	7(19.44 %)	
4	0(0.00 %)	2(3.85 %)		0(0.00 %)	1(2.78 %)	
Upper-right lung score			<0.001[b]			0.014[b]
0	19(55.88 %)	6(11.54 %)		12(52.17 %)	7(19.44 %)	

1	13(38.24 %)	34(65.38 %)		7(30.43 %)	16(44.44 %)	
2	0(0.00 %)	12(23.08 %)		3(13.04 %)	13(36.11 %)	
3	1(2.94 %)	0(0.00 %)		-	-	
4	1(2.94 %)	0(0.00 %)		1(4.35 %)	0(0.00 %)	
Middle-right lung score			<0.001[b]			0.146[b]
0	25(73.53 %)	18(34.62 %)		11(47.83 %)	9(25.00 %)	
1	8(23.53 %)	22(42.31 %)		7(30.43 %)	20(55.56 %)	
2	1(2.94 %)	11(21.15 %)		5(21.74 %)	6(16.67 %)	
3	0(0.00 %)	1(1.92 %)		0(0.00 %)	1(2.78 %)	
Bottom-right lung score			<0.001[b]			<0.001[b]
0	10(29.41 %)	0(0.00 %)		8(34.78 %)	0(0.00 %)	
1	14(41.18 %)	17(32.69 %)		9(39.13 %)	11(30.56 %)	
2	10(29.41 %)	27(51.92 %)		4(17.39 %)	17(47.22 %)	
3	0(0.00 %)	7(13.46 %)		1(4.35 %)	8(22.22 %)	
4	0(0.00 %)	1(1.92 %)		1(4.35 %)	0(0.00 %)	

a: Significant level, P<0.05

b: Fisher’s exact test.

Table S3 The appearing frequency of the selected radiomics features during 100-times bootstrapping.

Index	Feature name	Count
1	wavelet.HHH_glcm_Imc1	93
2	wavelet.LLH_glcm_InverseVariance	82
3	wavelet.HLH_glszm_SmallAreaEmphasis	77
4	wavelet.HLH_gldm_DependenceNonUniformityNormalized	76
5	original_glszm_SizeZoneNonUniformity	72
6	wavelet.HHL_glcm_Idn	70
7	original_firstorder_10Percentile	67
8	wavelet.LLL_glszm_SizeZoneNonUniformityNormalized	64
9	original_shape_Flatness	63
10	wavelet.LHL_firstorder_Skewness	47

Table S4 The appearing frequency of the selected radiomics features during 100-fold leave-group-out cross-validation (LGOCV).

Index	Feature name	Count
1	wavelet.HHH_glcm_Imc1	84
2	wavelet.LLH_glcm_InverseVariance	83
3	wavelet.HLH_glszm_SmallAreaEmphasis	74
4	wavelet.HHL_glcm_Idn	73
5	original_firstorder_10Percentile	71
6	original_glszm_SizeZoneNonUniformity	71
7	wavelet.HLH_gldm_DependenceNonUniformityNormalized	70
8	original_shape_Flatness	69
9	wavelet.LLL_glszm_SizeZoneNonUniformityNormalized	59
10	wavelet.LHL_firstorder_Skewness	43

Table S5 Estimation of the area under the ROC curve and the model overoptimism for the radiomics model using 100-times bootstrapping and 100-fold leave-group-out cross-validation (LGOCV) method.

Index	Model	Apparent AUC[a]	AUC SPLIT-Train[b] (mean, 95%CI)	AUC SPLIT-Test[c] (mean, 95%CI)	Average optimism[d]	Optimism-corrected AUC[e]
**1**	**BOOTSTRAP**	0.994(0.984-1.0)	1.0(0.999-1.0)	0.906(0.894-0.918)	0.094	0.9
**2**	**LGOCV**	0.994(0.984-1.0)	1.0(0.999-1.0)	0.903(0.893-0.914)	0.096	0.898

Note:

a. The AUC of predicting model developed in original training dataset.
b. The averaged model performance in the sub-training set among multiple-split dataset.
c. The averaged model performance in the sub-test set among multiple-split dataset.
d. The model's averaged optimism as the difference between the sub-training and sub-test set.
e. The corrected AUC by subtracting the average optimism from the apparent AUC.

Table S6 The ROC comparison between different models using Delong test.

**Model comparison**	**Z**	**P-value**
Train: Model A vs. Model B	4.8616	1.164e-06
Test: Model A vs. Model B	3.4784	0.0005044
Train: Model A vs. Model C	3.9943	6.487e-05
Test: Model A vs. Model C	3.1261	0.001772
Train: Model A vs. Model D	5.3341	9.6e-08
Test: Model A vs. Model D	3.9245	8.69e-05
Train: Model A vs. Model E	3.6101	0.000306
Test: Model A vs. Model E	3.2286	0.001244
Train: Model B vs. Model C	-2.9548	0.003129
Test: Model B vs. Model C	-1.0758	0.282
Train: Model B vs. Model D	2.2199	0.02642
Test: Model B vs. Model D	1.7362	0.08253
Train: Model B vs. Model E	-2.531	0.01137
Test: Model B vs. Model E	-1.0733	0.2831
Train: Model C vs. Model D	3.8847	0.0001025
Test: Model C vs. Model D	2.0286	0.0425
Train: Model C vs. Model E	-0.92225	0.3564
Test: Model C vs. Model E	-0.39297	0.6943
Train: Model D vs. Model E	-3.1182	0.00182
Test: Model D vs. Model E	-1.8642	0.06229

Statistically significant level: $P<0.05$

Table S7 The statistics of CT manifestations and signs features in Non-COVID-19(NC) and COVID-19 patients.

	Training set					Testing set				
CT signs	NC	COVID-19	Statistics	P-value[a]	Cramer's V	NC	COVID-19	Statistics	P-value	Cramer's V
Lesion distribution			58.083	<0.001[b]	0.792			22.440	<0.001[b]	0.596
Central	27(79.41%) (6.8)[c]	4(7.69%) (-6.8)				18(78.26%) (4.0)	9(25.00%) (-4.0)			
Peripheral	4(11.76%) (-7.3)	47(90.38%) (7.3)				2(8.70%) (-4.6)	25(69.44%) (4.6)			
Diffuse	1(2.94%) (0.3)	1(1.92%) (-0.3)				3(13.04%) (1.0)	2(5.56%) (-1.0)			
Non-specific	2(5.88%) (1.8)	0(0.00%) (-1.8)								
GGO involvement pattern			11.627	0.001 [b]	0.371			28.451	<0.001[b]	0.686
Patchy GGO	19(55.88%) (-3.4)	46(88.46%) (3.4)				7(30.43%) (-4.6)	32(88.89%) (4.6)			
Cluster-like GGO	13(38.24%) (3.2)	5(9.62%) (-3.2)				15(65.22%) (5.3)	1(2.78%) (-5.3)			
Combination of GGO and consolidation opacities	2(5.88%) (1.0)	1(1.92%) (-1.0)				1(4.35%) (-0.6)	3(8.33%) (0.6)			
Lobe predomination			3.867	0.157[b]				6.536	0.039[b]	0.358
Superior lobe	12(35.29%)	10(19.23%)				5(21.74%) (-1.4)	14(38.89%) (1.4)			
Inferior lobe	22(64.71%)	39(75.00%)				14(60.87%) (0.0)	22(61.11%) (0.0)			
Middle lobe	0(0.00%)	3(5.77%)				2(8.70%) (1.8)	0(0.00%) (-1.8)			
Balanced predomination						2(8.70%) (1.8)	0(0.00%) (-1.8)			
Lesion Contour			13.558	<0.001	0.397			3.059	0.08	
Shrinking	2(5.88%) (-3.7)	22(42.31%) (3.7)				4(17.39%)	14(38.89%)			
NonShrinking	32(94.12%) (3.7)	30(57.69%) (-3.7)				19(82.61%)	22(61.11%)			
Bronchial wall thickening			30.064	<0.001	0.591			10.09	0.003	0.414
No	18(52.94%) (-5.5)	52(100.00%) (5.5)				13(56.52%) (-3.2)	33(91.67%) (3.2)			
Yes	16(47.06%) (5.5)	0(0.00%) (-5.5)				10(43.48%) (3.2)	3(8.33%) (-3.2)			
Air bronchogram			3.997	0.046	0.216			0.348	0.555	
No	20(58.82%) (-2.0)	41(78.85%) (2.0)				17(73.91%)	24(66.67%)			
Yes	14(41.18%) (2.0)	11(21.15%) (-2.0)				6(26.09%)	12(33.33%)			
Tree-in-bud sign			20.363	<0.001	0.487			36.624	<0.001	0.788
No	18(52.94%) (-4.5)	49(94.23%) (4.5)				5(21.74%) (-6.1)	35(97.22%) (6.1)			
Yes	16(47.06%) (4.5)	3(5.77%) (-4.5)				18(78.26%) (6.1)	1(2.78%) (-6.1)			
Interlobular septal thickening			0.074	0.786				0.348	0.555	
No	27(79.41%)	40(76.92%)				17(73.91%)	24(66.67%)			
Yes	7(20.59%)	12(23.08%)				6(26.09%)	12(33.33%)			
Intralobular septal thickening			2.098	0.147				3.952	0.047	0.259

No	22(64.71%)	41(78.85%)			13(56.52%) (-2.0)	29(80.56%) (2.0)		
Yes	12(35.29%)	11(21.15%)			10(43.48%) (2.0)	7(19.44%) (-2.0)		
Pleura effusion				1.0[b]			0.0	0.39
No	33(97.06%)	50(96.15%)			22(95.65%)	36(100.00%)		
Yes	1(2.94%)	2(3.85%)			1(4.35%)	0(0.00%)		
Lesion attenuation			2.451	0.117			3.838	0.05
GGO	7(20.59 %)	19(36.54 %)			2(8.70 %)	11(30.56 %)		
GGO with consolidation	27(79.41 %)	33(63.46 %)			21(91.30 %)	25(69.44 %)		

a: significant level, P<0.05

b: Fisher’s exact test.

c: The adjusted residual. The absolute value of adjusted residual larger than 2.58 was considered to be significant.

Supplementary Method

1. Image analysis based on Lung Kit software

In the current study, the radiomics features and several quantitative derived CT features were extracted from Lung Kit software (Version 2.2, GE Healthcare). The software is one commercialized software only for scientific research and the analysis module for COVID-19 involves the four-step data processing flow which is described as follows.

(1) Image loading and user-defined image preprocessing: In the current research, all the images were anonymized with patient information and loaded into LK2.2 software as nifty format. All the images were firstly resampled into isotropic voxel size of 1 mm*1 mm*1 mm using trilinear interpolation. After interpolation into isotropic voxel, the image's intensity values were rounded to the nearest integer HU value. The low-pass Gaussian filter with $\sigma = 0.5$ was then conducted to increase the reproducibility of the radiomics features.

(2) The automatic segmentation of lung lobes and pneumonia lesion: The five lung lobes were segmented based on deep learning algorithm Dense V-networks [*Gibson E, Giganti F, Hu Y, Bonmati E, Bandula S, Gurusamy K, Davidson B, Pereira SP, Clarkson MJ, Barratt DC. Automatic Multi-Organ Segmentation on Abdominal CT With Dense V-Networks. IEEE Trans Med Imaging. 2018 Aug;37(8):1822-1834. doi: 10.1109/TMI.2018.2806309. Epub 2018 Feb 14. PMID: 29994628; PMCID: PMC6076994.*], including 2 lobes in left lung (upper, bottom) and 3 lobes in right lung (upper, middle, bottom). Based on the lung lobes segmentation, the pneumonia lesions were detected and volume of interest (VOI) was segmented as a whole. The machine-human collaboration was applied to guarantee correct segmentation. The margin of the VOI was checked and manually adjusted by an experienced thoracic radiologist (SK. P, a radiology attending doctor with 7 years' experience in interpreting chest CT images) and the obviously swollen blood vessels

involved in the lesion were excluded, if necessary. All the automatically segmented or manually adjusted VOIs were checked by a senior radiologist (H. P [a thoracic radiologist with 28 years' experience]) to reach consensus. The distributed lesions were considered as a whole VOI in the following analysis steps.

(3) Quantization of pneumonia lesion: the volume of each segmented lung lobe and pneumonia lesion were firstly calculated. The lesion volume ratio and lesion component analysis were conducted as follows.

For lesion volume ratio analysis, the lesion volume ratio in five lung lobes was respectively calculated automatically by LK2.2 after lesion VOI was delineated. The lesion ratio in each lung lobe was scored from 0 to 5 which was defined according to the volume ratio involved: 0, no lesion; 1, ≤5%; 2, 6%-25%; 3, 26%-49%; 4, 50%-75%; 5, >75% [*Pan F, Ye T, Sun P, Gui S, Liang B, Li L, Zheng D, Wang J, Hesketh RL, Yang L, Zheng C. Time Course of Lung Changes at Chest CT during Recovery from Coronavirus Disease 2019 (COVID-19). Radiology. 2020 Jun;295(3):715-721. doi: 10.1148/radiol.2020200370. Epub 2020 Feb 13. PMID: 32053470; PMCID: PMC7233367.*].

For lesion component analysis, the part solid component was defined as the lesion with CT value ≤-200 HU while the parts with CT value >-200 HU were defined as solid component. And the volume ratio of each kind of lesion component in the left and right lung was derived automatically.

(4) Radiomics feature extraction based on the lesion VOIs: The LK2.2 software carried open source of Python package Pyradiomics v2.2 (https://pyradiomics.readthedocs.io/en/latest/index.html) and provide user interface to set the parameters for feature extraction. In the current study, following the CT value discretized with binWidth = 25HU, a total of 851 radiomics features were extracted from segmented pneumonia VOIs *[Larue RTHM, van Timmeren JE, de Jong EEC, Feliciani G, Leijenaar RTH, Schreurs WMJ,*

Sosef MN, Raat FHPJ, van der Zande FHR, Das M, van Elmpt W, Lambin P. Influence of gray level discretization on radiomic feature stability for different CT scanners, tube currents and slice thicknesses: a comprehensive phantom study. Acta Oncol. 2017 Nov;56(11):1544-1553. doi: 10.1080/0284186X.2017.1351624. PMID: 28885084.].

A total of 851 extracted radiomics features were categorized into three groups: first-order features, textural features, and transformed features. There were 32 first-order features consisting of 18 intensity statistical features and 14 morphological features. Among 75 textural features, there were 24 Gray Level Co-occurrence Matrix (GLCM), 16 Gray Level Run Length Matrix (GLRLM), 16 Gray Level Size Zone Matrix (GLSZM), 14 Gray Level Dependence Matrix (GLDM) and 5 Neighboring Gray Tone Difference Matrix (NGTDM) features. For the transformed images, first-order wavelet transform decomposed the ROI into 8 sub-VOIs by using either a high- or low-pass filter in three dimensional directions, including high–high–high, high–high–low, high–low–low, high–low–high, low–high–low, low–high–high, low–low–high, and low–low–low. The same set of texture features (18 intensity statistical features, 24 GLCM features, 16 GLRLM, 16 GLSZM, 14 GLDM, and 5 NGTDM) were calculated based on these wavelet-transformed images and 744 wavelet features were obtained finally.

The extracted quantitative CT features and radiomics features were then analyzed and modeled based on the method described in the main text.

2. The method for the 100-times bootstrapping and LGOCV

We conducted 100-times bootstrapping and 100-fold leave-group-out cross-validation (LGOCV) with the proportion of data in the sub–training sets (60%), to measure the reliability of the radiomics features involved in the final model and the model overoptimism. The reliability of the radiomics features was measured by the appearing frequency during multiple splits of training and test sets. While the model overoptimism was verified by the mean AUC in the multiple-split training and

test sets.

2.1 100-times bootstrapping method

Step 1: Apparent performance. Taking the model's AUC performance of predicting model developed in the original whole training dataset as the apparent performance.

Step2: BOOTSTRAP sample splitting. The whole original training dataset was repeatedly split into bootstrapped training and test sets. The bootstrapped training set with the same sample size as original training dataset was constructed by sampling with replacement from the original sample. The out-of-bag samples constructed the test set in each bootstrap.

Step3: BOOTSTRAP model establishment. Starting features were features used to establish radiomics model based on the original training dataset. And these features were further selected by backward stepwise logistic regression method with minimum AIC (the same as the modeling method in the manuscript) in each bootstrapped training set. The logistic regression model established from the bootstrapped training set was respectively tested in the "out-of-bag" test set. The AUC were obtained for bootstrapped training and test set in each bootstrap loop.

Step4: Model optimism among bootstrap. Calculate the model optimism as the difference between the AUC of bootstrapped training set and the test set.

Step5: The optimism-corrected performance. Repeating Step 2 to Step 4 for 100 times. The appearing frequency of each feature and the average optimism were calculated and recorded. Subtract the value from the apparent performance in step 1 and obtain an optimism-corrected performance.

2.2 100-fold leave-group-out cross-validation (LGOCV) method

Step 1: Apparent performance. Taking the model's AUC performance of predicting model developed in the original whole training dataset as the apparent performance.

Step2: Multiple splitting of training and test sets. The whole dataset was repeatedly split into training and test sets. A proportion of data in the sub–training sets was set as 60% which was the same as the randomized stratification ratio of 3:2 in the original whole dataset. The rest 40% data were taken as test set in each loop. Such group splitting was repeated 100 times.

Step3: LGOCV model establishment. Starting features were features used to establish radiomics model based on the original training dataset. And these features were further selected by backward stepwise logistic regression method with minimum AIC (the same as the modeling method in the manuscript) in each LGOCV training set. The logistic regression model established from the LGOCV training set was respectively tested in the LGOCV test set. The AUC were obtained for LGOCV training and test set in each loop.

Step4: Model optimism among multiple splitting. Calculate the model optimism as the difference between the AUC of LGOCV training set and test set.

Step5: The optimism-corrected performance. Repeating Step 2 to Step 4 for 100 times. The appearing frequency of each feature and the average optimism were calculated and recorded. Subtract the value from the apparent performance in step 1 and obtain an optimism-corrected performance.
