# Supplementary material for: Quantitative CT imaging features for COVID-19 evaluation: The ability to differentiate COVID-19 from non- COVID-19 (highly suspected) pneumonia patients during the epidemic period
Source: PLoS One. 2022 Jan 13;17(1):e0256194. doi: 10.1371/journal.pone.0256194 (PMC8758079; doi:10.1371/journal.pone.0256194)
Supplement: Attachment — Submitted filename: Response to Reviewers_20210625.docx [file pone.0256194.s003.docx]

**Dear Dr. Gayle E. Woloschak,**

We are very pleased to learn again from your letter about revision for our manuscript (PONE-D-20-25966R1). Thank you very much for providing us the reviewers’ further suggestion and the opportunity for us to resubmit our revised manuscript. We have revised the manuscript according to the comments from the reviewers. Our answers to the reviewers’ comments are described as follows, with revision tracks in the revised manuscript and supplementary material.

We hope that the revised manuscript could be considered acceptable for publication. We are looking forward to hearing from you.

Sincerely,

Thank you and all the best regards,

Dr. Hong Pu, M.D, PHD,

Professor and Director,

Department of Radiology, Sichuan Academy of Medical Sciences & Sichuan Provincial People's Hospital, University of Electronic Science and Technology of China, Chengdu, China.

**PONE-D-20-25966R1
Quantitative CT imaging features for COVID-19 evaluation: the ability to differentiate COVID-19 from Non- COVID-19 (Highly Suspected) pneumonia patients during the epidemic period**

**PLOS ONE**

**Answer to Reviewer #1:**

**Reviewer #1:** The response to my critiques for the previous submission looks adequate and appropriate. However, the details of “Lung Kit” software, in the response letter, better be in either the manuscript’s main text or at least in the supplementary material. It is still not clear how others could reproduce the results without knowing that it can be obtained for research.

**Response:**

We sincerely thank the reviewer again for giving us valuable suggestion to perfect the method description and make it friendly to our reader. Following the reviewer’s suggestion, we have supplemented the detailed procedure for image analysis based on the Lung Kit software in the supplementary method part. We also copied the context here for reviewer’s reading convenience.

**Supplementary Method**

1. **Image analysis based on Lung Kit software**

In the current study, the radiomics features and several quantitative derived CT features were extracted from Lung Kit software (Version 2.2, GE Healthcare). The software is one commercialized software only for scientific research and the analysis module for COVID-19 involves the four-step data processing flow which is described as follows.

**(1) Image loading and user-defined image preprocessing:** In the current research, all the images were anonymized with patient information and loaded into LK2.2 software as nifty format. All the images were firstly resampled into isotropic voxel size of 1 mm*1 mm*1 mm using trilinear interpolation. After interpolation into isotropic voxel, the image’s intensity values were rounded to the nearest integer HU value. The low-pass Gaussian filter with σ = 0.5 was then conducted to increase the reproducibility of the radiomics features.

**(2) The automatic segmentation of lung lobes and pneumonia lesion:** The five lung lobes were segmented based on deep learning algorithm Dense V-networks [*Gibson E, Giganti F, Hu Y, Bonmati E, Bandula S, Gurusamy K, Davidson B, Pereira SP, Clarkson MJ, Barratt DC. Automatic Multi-Organ Segmentation on Abdominal CT With Dense V-Networks. IEEE Trans Med Imaging. 2018 Aug;37(8):1822-1834. doi: 10.1109/TMI.2018.2806309. Epub 2018 Feb 14. PMID: 29994628; PMCID: PMC6076994.*], including 2 lobes in left lung (upper, bottom) and 3 lobes in right lung (upper, middle, bottom). Based on the lung lobes segmentation, the pneumonia lesions were detected and volume of interest (VOI) was segmented as a whole. The machine-human collaboration was applied to guarantee correct segmentation. The margin of the VOI was checked and manually adjusted by an experienced thoracic radiologist (SK. P, a radiology attending doctor with 7 years’ experience in interpreting chest CT images) and the obviously swollen blood vessels involved in the lesion were excluded, if necessary. All the automatically segmented or manually adjusted VOIs were checked by a senior radiologist (H. P [a thoracic radiologist with 28 years’ experience]) to reach consensus. The distributed lesions were considered as a whole VOI in the following analysis steps.

**(3) Quantization of pneumonia lesion**: the volume of each segmented lung lobe and pneumonia lesion were firstly calculated. The lesion volume ratio and lesion component analysis were conducted as follows.

For lesion volume ratio analysis, the lesion volume ratio in five lung lobes was respectively calculated automatically by LK2.2 after lesion VOI was delineated. The lesion ratio in each lung lobe was scored from 0 to 5 which was defined according to the volume ratio involved: 0, no lesion; 1, ≤5%; 2, 6%-25%; 3, 26%-49%; 4, 50%-75%; 5, >75% [ *Pan F, Ye T, Sun P, Gui S, Liang B, Li L, Zheng D, Wang J, Hesketh RL, Yang L, Zheng C. Time Course of Lung Changes at Chest CT during Recovery from Coronavirus Disease 2019 (COVID-19). Radiology. 2020 Jun;295(3):715-721. doi: 10.1148/radiol.2020200370. Epub 2020 Feb 13. PMID: 32053470; PMCID: PMC7233367.*].

For lesion component analysis, the part solid component was defined as the lesion with CT value ≤-200 HU while the parts with CT value >-200 HU were defined as solid component. And the volume ratio of each kind of lesion component in the left and right lung was derived automatically.

**(4) Radiomics feature extraction based on the lesion VOIs:** The LK2.2 software carried open source of Python package Pyradiomics v2.2 (https://pyradiomics.readthedocs.io/en/latest/index.html) and provide user interface to set the parameters for feature extraction. In the current study, following the CT value discretized with binWidth = 25HU, a total of 851 radiomics features were extracted from segmented pneumonia VOIs *[Larue RTHM, van Timmeren JE, de Jong EEC, Feliciani G, Leijenaar RTH, Schreurs WMJ, Sosef MN, Raat FHPJ, van der Zande FHR, Das M, van Elmpt W, Lambin P. Influence of gray level discretization on radiomic feature stability for different CT scanners, tube currents and slice thicknesses: a comprehensive phantom study. Acta Oncol. 2017 Nov;56(11):1544-1553. doi: 10.1080/0284186X.2017.1351624. PMID: 28885084.].*

A total of 851 extracted radiomics features were categorized into three groups: first-order features, textural features, and transformed features. There were 32 first-order features consisting of 18 intensity statistical features and 14 morphological features. Among 75 textural features, there were 24 Gray Level Co‐occurrence Matrix (GLCM), 16 Gray Level Run Length Matrix (GLRLM), 16 Gray Level Size Zone Matrix (GLSZM), 14 Gray Level Dependence Matrix (GLDM) and 5 Neighboring Gray Tone Difference Matrix (NGTDM) features. For the transformed images, first-order wavelet transform decomposed the ROI into 8 sub-VOIs by using either a high- or low-pass filter in three dimensional directions, including high–high–high, high–high–low, high–low–low, high–low–high, low–high–low, low–high–high, low–low–high, and low–low–low. The same set of texture features (18 intensity statistical features, 24 GLCM features, 16 GLRLM, 16 GLSZM, 14 GLDM, and 5 NGTDM) were calculated based on these wavelet-transformed images and 744 wavelet features were obtained finally.

The extracted quantitative CT features and radiomics features were then analyzed and modeled based on the method described in the main text.

**Answer to Reviewer #2:**

**Reviewer #2:** The authors satisfactorily addressed all the comments raised in the initial review and made significant changes to the manuscript accordingly.
**Response:**

We sincerely thank the reviewer again for the kind patience and valuable suggestion during the peer-review process.
